# Omega-3 fatty acids does not affect physical activity and body weight in primary school children – a double-blind randomized placebo-controlled trial

**DOI:** 10.1038/s41598-018-31229-4

**Published:** 2018-08-24

**Authors:** V. Svensson, E. Johansson, M. Fischer, S. L. Deng, M. Hagströmer, P. Danielsson

**Affiliations:** 1 Karolinska Institutet, Division of Pediatrics, Department of Clinical Science, Intervention and Technology, Stockholm, Sweden; 2 Karolinska Institutet, Department of Neurobiology, Care Sciences and Society, Division of Physiotherapy, Stockholm, Sweden; 3 Wuhan University of Technology, Wuhan Shi, China

## Abstract

It was hypothesized that supplementation of omega-3 fatty acids could increase physical activity (PA) levels, where traditional interventions often fail. The aim of this double-blind, randomized, placebo-controlled trail was to evaluate the effects of 15-week administration of omega-3 fatty acids on objectively measured PA and relative body weight in 8–9 year-old children. The children were randomly assigned to supplementation of omega-3 fatty acids or placebo. Primary outcome was change in PA counts per minute (cpm), and secondly change in body mass index standard deviation score (BMI SDS). Covariance models were applied adjusting for age, gender, weight status, PA and intervention season. Compliance was controlled for by analyzing fatty acid composition in plasma. The intention to treat population consisted of 362 children (omega-3 n = 177, placebo n = 185). No significant effects of omega-3 fatty acids on PA or relative body weight were observed. In covariance models no effects were observed by gender, weight status or change in PA (all p > 0.05), but inactive children increased their PA more than children classified as active at baseline (p < 0.05).

## Introduction

Obesity in childhood has immediate consequences on health, including hyperlipidemia, hypertension, abnormal glucose tolerance, and psychosocial health effects^1,2^. Since treatment of obesity is difficult^3^, prevention has been pronounced as a public health priority. It is well known that dietary intake affects body weight^4^. The importance of physical activity (PA) for obesity prevention is under debate but it is possible that PA during childhood can be protective against obesity^5^. More importantly, PA prevents and reduces obesity-related comorbidities such as diabetes and cardiovascular diseases^6,7^. Several attempts have been made to increase PA in school-aged children but this has proven difficult to affect using traditional interventions^8^.

There is considerable evidence that a high intake of omega-3 fatty acids has beneficial effects on health by, for example, favorably improving triglyceride levels, affecting blood clotting and vasoconstriction, having inflammatory properties and reducing body fat^9^. The mechanisms by which omega-3 fatty acids reduce body fat are not well understood, but altered gene expression, favoring increased fat oxidation in adipose, liver, cardiac, intestinal and skeletal muscle tissue and reduced fat deposition in adipose tissue have been suggested^10^. In rodents and in humans, omega-3 fatty acids stimulate β-oxidation, and inhibit fatty acid synthesis and very-low density lipoprotein secretion^11^. Also, an enhancement of post-prandial satiety may lead to reduced food intake and contribute to a reduction in body fat^10^. In this context, omega-3 supplementation may have potential anti-obesity properties. The mechanisms by which omega-3 could influence PA is unclear but improvement of bodily capacity may influence the experience of PA in a more positive direction and by that affecting PA behaviors. Omega-3 fatty acids affect muscle mass and strength^12^ and it has been found to increase performance in cancer patients^13^. Only one study has explored the associations between omega-3 fatty acids and PA in humans^14^. In that study, it was found that adult patients with pancreatic cancer increased their total energy expenditure and physical activity level after eight weeks of omega-3 supplementation.

In 2008, a pilot study was carried out in Stockholm, Sweden. The aim was to evaluate if motivational interviewing (MI) could increase PA in children aged 6–10 years with low initial PA levels. Children were randomly assigned to one of four treatments, comprising: supplementation of omega-3 and MI; placebo and MI; supplementation of omega-3 only; or placebo only. No effect of MI was found on PA, but children in the omega-3-group did increase their PA levels (unpublished data). In this study, we aimed to confirm or reject these results on a larger sample of children.

The objective of this study was to evaluate the effect of 15 weeks’ administration of 732 mg/day of omega-3 fatty acids (556 mg EPA, 176 mg DHA) on PA and relative body weight in children aged 7–9 years.

## Material and Methods

### Study design and participants

This double-blind, randomized, placebo-controlled trail examined 7–9 year-old children during autumn 2011 and spring 2012, recruited from public elementary schools in Stockholm, Sweden. After inviting a broad range of elementary schools from all over Stockholm, 18 agreed to participate. Children without diseases or syndromes that affect hormonal levels other than well-controlled hypothyroidism were included. A total of 423 children (boys, n = 209; girls, n = 214) were eligible and enrolled in this study. Of the 423 children, 204 were allocated to the omega-3 fatty acids group and 219 to the placebo group. Written informed consent was obtained from each child and their parents or guardians. The study was approved by the Regional Ethical Review Board in Stockholm, Sweden, and the methods were carried out in accordance with this approval (Dnr. 2011/1176-31/2). The study has also been registered in Clinical Trials Registry (clinicaltrials.gov Date: 25/03/2011, ID: NCT01087411).

### Procedures

Children were randomly assigned to 15 weeks of omega-3 fatty acid supplementation or placebo (rapeseed oil) via a double-blind procedure. Information about the allocation was concealed until the follow up data were collected. Once participants were randomized, baseline measures (anthropometrics, background data and PA) were taken and repeated after the intervention period (at week 15). All the measures took place during normal school hours in a quiet room within the school, and were performed by two trained researchers, blinded to the randomization. At the beginning of the trial school staff and parents were given instructions for dispensing capsules and a diary to record capsule consumption. A set of standard written materials was provided to the parents along with nutritional counseling about healthy eating. To determine compliance, levels of omega-3 fatty acids EPA and DHA (fractions (%) of total fatty acids) were measured through blood samples before and after the intervention in a subgroup (33 and 39 children in the intervention and placebo groups, respectively). The blood samples were collected after an overnight fast by a research nurse with extensive experience of blood sampling in children. The EPA and DHA levels in the lipid fractions in plasma were analyzed using the gas-liquid chromatography (GLC) technique.

### Anthropometric measurements

Height and weight were measured using standard clinical procedures with a transportable Harpenden stadiometer and a digital scale (Tanita BWB 800S, Tanita, Tokyo, Japan). Height was measured to the nearest 0.01 m and weight to the nearest 0.1 kg. All children were measured in the morning wearing underwear only. Body mass index (BMI) was calculated as weight in kilograms divided by height in meters squared and was expressed in kg/m^2^. Body mass index standard deviation score (BMI SDS), a measure of relative weight in children that accounts for difference in BMI by age and gender, was calculated using a reference standard^15^. Children were categorized as having normal weight, overweight, or obesity, as defined by the International Obesity Task Force (IOTF)^16^.

### Measurements of PA

The Actigraph GT3X+ accelerometer (Actigraph, Pensacola, Florida, USA) was used to monitor PA during seven consecutive days. Children were asked to wear the accelerometer on the non-dominant wrist without removing it except for during swimming and bathing. Data were collected at 30 Hz and summarized over 10-second epochs. To exclude sleep time, the hours between 9 pm and 8 am were removed prior to data analysis, based on a previous study exploring sleep patterns among Swedish 6–10 year olds^17^. Ten consecutive minutes with zero counts were considered as non-wear time and was removed from the analysis. Children with at least 4 days of accelerometer data, with at least 10 hours of registration per day were included^18^.

The intervention season was divided into fall-winter or winter-spring. The children had repeated PA measurements within either fall-winter (n = 144), or winter-spring (n = 218), and difference in mean PA level between intervention seasons was assessed. Outcome measures were average PA counts per minute (cpm) for the vertical axis. Quartiles of PA level at baseline and follow-up were also calculated. PA level at baseline was categorized into; physically inactive (Q_1_) or physically active (Q_2–4_). PA change from baseline was categorized into; PA decreased (quartile of PA follow up - quartile of PA baseline <0), PA unchanged (quartile PA follow up – quartile PA baseline = 0) or PA increased (quartile of PA follow up - quartile of PA baseline >0).

### Intervention

The omega-3 fatty acids were administered in soft gel fruit-flavored capsules, provided by Midsona AB. The capsules were chewable and did not need to be swallowed whole. As in the previous pilot study, the daily dose consisted of four capsules, in total 732 mg of omega-3 fatty acids (556 mg EPA, 176 mg DHA), 60 mg of omega-6 (fatty acid GLA) and 9.6 mg of vitamin E in natural form, i.e. alpha-tocopherol (antioxidant). The placebo group received capsules, containing rapeseed oil, identical in appearance to omega-3 fatty acids tablets. The content of a full day’s dose was equivalent to about 10 kcal, which was deemed not to affect a child’s energy balance significantly.

### Statistical analysis

Intent-to-treat (ITT) analyses were carried out, including all participants who took at least one dose of omega-3 supplement or placebo and had baseline data on weight, height and PA. The last observation carried forward (LOCF) method was applied for missing PA and BMI SDS at follow-up, after controlling to ensure that missing data did not differ significantly between groups. Comparisons between the omega-3 and the placebo groups were examined using multivariate analysis of covariance (ANCOVA). Analyses of subgroups were conducted by gender, intervention season, baseline weight status, baseline PA level, and change in PA. Differences in EPA and DHA levels between baseline and follow-up were assessed with paired t-test. Sample size (with 80% power and alfa 0.05) was calculated based upon previous findings in the absence of references for expected variance. With data at hand today the sample size needed to detect a 10% change in PA between groups varied from 52 (26 per group) to 102 (51 per group).

The primary outcome variable was change in PA level from baseline to follow up, calculated by subtracting baseline PA cpm from PA cpm at follow-up. The fixed effects model was used for the main analysis and tested for potential intervention effects on PA level from: intervention group; sex; intervention season (fall-winter/winter-spring); and weight status at baseline. Age and PA cpm at baseline were included as covariates. Differences in PA change were also assessed by PA quartile at baseline (physically inactive (Q_1_) vs. physically active (Q_2–4_)).

The second outcome was change in BMI SDS from baseline to follow-up, calculated by subtracting baseline from follow-up BMI SDS. The fixed effects analysis tested for potential intervention effects on BMI SDS from: intervention group; intervention season; sex; baseline weight status; and change in PA (decreased, unchanged, increased). Baseline age and BMI SDS were included as covariates. Differences between the two groups in PA cpm and anthropometric measures at baseline were assessed with the independent samples t-test. PA differences in intervention seasons between the two groups were tested with univariate ANOVA. The categorical variables were analyzed using chi-square tests. A p-value < 0.05 was defined as the level of significance. Statistical analyses were performed using the software SPSS statistics (Version 17.0, 2008, SPSS Inc., Chicago, IL, USA).

## Results

### Baseline data

A total of 423 children were randomized to omega-3 and placebo. Sixty-one children were excluded from the ITT population, due to lack of baseline anthropometric measures (n = 10) or PA data (n = 51). The ITT population consisted of 362 children (omega-3 supplement n = 177, placebo n = 185). The mean age was 8.5 (SD 0.3) years and the proportion of boys was 50% (n = 181). Average BMI SDS was 0.3 (SD 1.2) and 81% had a normal weight, 14% had overweight and 5% had obesity. The children accumulated on average 2492 (SD 413) PA cpm. Baseline demographics, anthropometric measures, PA levels, and percentage sedentary time in the ITT population are shown in Table 1. The two groups did not differ according to any other demographic or PA variable, except for a significantly lower BMI SDS at baseline in the omega-3 group, compared with the placebo group (0.2 (SD 1.2) vs. 0.5 (SD 1.2), p = 0.035). In both groups, PA level in winter-spring was significantly higher than that in fall-winter. In the subgroup analysis, no differences between the groups were found in fractions of EPA and DHA (EPA 0.2 (SD 0.4)%, DHA 4.0 (SD 0.7)%) at baseline. The group receiving omega-3 increased their levels of EPA to 3.5 (SD 1.3) and DHA to 5.5 (SD 1.0), while levels in the placebo group remained the same (p < 0.001).

**Table 1:** Baseline characteristics in the omega-3 supplement and placebo group; Intention to treat population (ITT).

	Placebo		Omega-3 FAs		Total		*p*
	n	Mean (SD)	n	Mean (SD)	n	Mean (SD)	
Sex(n, %)							0.92
Boys	93	50%	88	50%	181	50%	
Girls	92	50%	89	50%	181	50%	
Weight status (n, %)					362		0.61
Normal weight	146	79%	147	83%	293	81%	
Overweight	29	16%	22	12%	51	14%	
Obesity	10	5%	8	5%	18	5%	
Age	185	8.5 (0.3)	177	8.5 (0.3)	362	8.5 (0.3)	0.57
Weight	185	30.9 (6.0)	177	29.8 (0.4)	362	30.4 (0.3)	0.08
Height	185	1.34 (0.06)	177	1.34 (0.06)	362	1.34 (0.03)	0.43
BMI	185	17.1 (2.3)	177	16.6 (2.1)	362	16.9 (2.2)	0.06
BMI SDS*	185	0.47 (1.15)	177	0.21 (1.16)	362	0.34 (0.06)	0.04
PA mean cpm	185	2478 (404)	177	2507 (424)	362	2492 (413)	0.49
PA Q1	51	2005 (172)	38	1908 (236)	89	1969 (22)	0.03
PA Q2	45	2338 (84)	44	2360 (88)	89	2359 (8)	0.24
PA Q3	40	2609 (76)	52	2643 (80)	92	2637 (9)	0.04
PA Q4	49	2989 (213)	43	3022 (213)	92	3010 (22)	0.45
Intervention season**							
Winter-Spring (PA cpm)	115	2421 (377)	104	2461 (391)	219	2439 (384)	0.45
Fall-Winter (PA cpm)	70	2569 (429)	73	2573 (461)	143	2572 (443)	0.95

BMI = body mass index; BMI SDS = BMI standard deviation score;

PA = physical activity; cpm = counts per minute; Q = quartile.

*p < 0.05 vs placebo; **p < 0.01 vs seasons.

### Change in PA

In both groups, PA levels increased significantly over the intervention period (145 (SD 383) cpm, p < 0.001). No significant effect of the intervention on PA cpm was observed in adjusted analysis (p = 0.43; Table 2). Furthermore, no significant effect of the intervention on PA cpm was found in any of the subgroups (all p > 0.05, Table 2). Change in PA did not differ by gender or between normal weight and overweight/obese children. Children who were classified as inactive at baseline increased their PA levels significantly more than children who were classified as active at baseline (change in mean cpm 224 vs. 119; p < 0.05). The increase in PA was also significantly higher during the winter-spring period compared with the fall-winter period (change in mean cpm 229 vs 143; p < 0.01).

**Table 2:** Change in physical activity counts per minute (PA cpm).

	Placebo		n-3 FAs		*p*
	n	Mean (SD)	n	Mean (SD)	
ΔPA	185	162 (402)	177	130 (365)	0.43
Subgroup: sex					
Girls	92	132 (350)	89	158 (324)	0.61
Boys	93	191 (448)	88	102 (402)	0.16
Subgroup: intervention season					
Winter-Spring	115	220 (411)	104	180 (346)	0.44
Fall-Winter	70	67 (371)	73	58 (381)	0.89
Subgroup: WS at baseline					
OW/OB	39	166 (348)	30	186 (352)	0.81
NW	146	161 (417)	147	118 (368)	0.36

LS = least squares; PA = physical activity;

WS = weight status; OW/OB = Overweight/obesity; NW = Normal weight.

### Change in BMI SDS

BMI SDS did not change significantly over the intervention period (p = 0.91). No significant differences in change in BMI SDS were observed according to sex, BMI SDS (normal weight or overweight/obesity), intervention season or activity level at baseline or follow-up. There was no significant effect of the intervention on change in BMI SDS in adjusted analyses (p = 0.97, Table 3). In subgroup analyses, no significant effects of the intervention on BMI SDS change were observed (all p > 0.05, Table 3).

**Table 3:** Change in BMI SDS.

	Placebo		n-3 FAs		*p*
	n	Mean (SD)	n	Mean (SD)	
ΔBMI SDS	185	0 (0.41)	177	0.01 (0.32)	0.97
Subgroup: WS at baseline					
Overweight/obesity	39	−0.03 (0.27)	30	0 (0.18)	0.52
Normal weight	146	−0.03 (0.27)	147	0.01 (0.34)	0.79
Subgroup: sex					
Girls	92	0.02 (0.34)	89	0 (0.30)	0.79
Boys	93	−0.03 (0.47)	88	0.01 (0.34)	0.86
Subgroup: PA changed					
PA decreased	75	0.06 (0.31)	70	−0.04 (0.35)	0.98
PA unchanged	82	−0.09 (0.46)	74	0.04 (0.30)	0.33
PA increased	28	0.06 (0.47)	33	0.06 (0.29)	0.76
Subgroup: PA status at baseline					
Inactive	51	0 (0.32)	38	0.04 (0.32)	0.69
Active	134	−0.01 (0.44)	139	0 (0.32)	0.37

PA = physical activity; WS = weight status.

## Discussion

The aim of this study was to explore whether omega-3 fatty acid supplementation affects PA or BMI SDS in healthy 7–9 year-old children. No significant effect on either PA or BMI SDS was identified. The study was based upon results from a previous pilot study performed by our group where we found effects of omega-3 supplementation on PA. The possible mechanisms linking omega-3 with PA can only be speculated upon. It is possible that the favorable effects of omega-3 on bodily function increases the appreciation of PA and by that affect the behavior^12,13^. In this study, children in both the intervention and control group increased their activity levels over the intervention period. This effect is likely due to seasonal variation. Swedish children are more active during summer time, when daylight and outdoor temperature facilitates outdoor play^19^. One important finding was, however, that children with the lowest initial levels of PA increased their PA significantly more that those with higher levels. This result is commensurate with results from a previous pilot study where children with low initial PA levels increased their PA irrespective of omega-3 supplementation. It could also be an effect of regression to the mean^20^. To our knowledge, only one other previous study has examined the effect of omega-3 on PA in humans^14^. In that study, where energy expenditure and physical activity level were assessed using doubly-labeled water, it was found that omega-3 supplementation increased PA in a small sample of patients with advanced pancreatic cancer.

Accumulating evidence from epidemiological and clinical studies suggests that an increased intake of omega-3 fatty acids can decrease the degree of obesity, enhance weight loss^21^, and decrease body fat mass^22^. Also, in children, lower levels of omega-3 fatty acids have been associated with increased body weight^23,24^. Contrary to these studies, omega-3 supplementation had no effect on BMI SDS in this study. The effect of omega-3 supplementation can vary by dosage, duration, sex and age^25^. The dose of omega-3 that was used in the present study is comparable to those used in most other trials of omega-3 fatty acids for child behavior^26,27^. It was also the exact same dose as had been used in the previous pilot study where we found an impact on PA. According to the Nordic Nutrition Recommendations (NNR), the recommended intake of omega-3 fatty acids for children and adults is ≥1 E%. For eight-year-old children that corresponds to at least 2 grams per day^28^. The dosage in this study (732 mg/day) is considerably lower than these recommendations and it is possible that the dose was too low to achieve an intervention effect. However, in addition to the supplementation provided, children are expected to ingest a substantial amount from food. A national survey found the average intake of omega-3 Swedish children 8–9 years to be 1,3 g/day^29^. Together with the supplementary dose of omega-3 given in this study, children were assumed to reach the recommendations of intake in this age group. Also, when this study was conducted, a higher dosage had not been sufficiently studied in children <12 years of age. Another possible reason for the lack of intervention effect is that the duration of the intervention was too short. In a meta-analysis, where most included studies had an intervention length of 2–3 months, a favorable effect on body weight was observed in adults^21^. In children, 12 weeks of omega-3 supplementation had a small but significant effect on BMI^26^. In accordance with this study, some previous studies have failed to find any effect of omega-3 supplementation on body composition in children^30–32^, indicating a need for further studies on this population. In comparison, our intervention was longer.

Strengths of the study are that the included participants were representative of the normal Swedish population regarding the proportion of with normal weight, overweight and obesity. They were also recruited from schools across a variety of socioeconomic areas in Stockholm County. To ensure compliance, blood levels of DHA and EPA were analyzed and found to differ significantly between the omega-3- and the placebo group throughout the intervention period. This indicates that the soft gel fruit-flavored capsule suits a pediatric population well as an omega-3 substitute. PA was measured objectively with accelerometry and anthropometric measurements were taken by trained researchers, blinded to the randomization.

Some limitations should be acknowledged. First, the participating children may have been particularly interested in the study, potentially causing a selection bias. It is possible that the study results are not generalizable to other populations. Secondly, higher BMI has been associated with lower blood levels of DHA after omega-3 supplementation^33^. Therefore, it is possible that higher baseline BMI SDS in the placebo group led to lower levels of DHA in plasma and moderated the impact on PA. Third, during the intervention period, children did not have energy restricted or controlled diets, which may have confounded the outcome^34^. Most studies in animals and humans, which have shown effects of omega-3 fatty acids on body weight and/or body composition, indicate that these effects are independent of energy intake. One study showed, however, that weight loss achieved through caloric restriction was enhanced when fish oil was included in the diet^10^. Last, as mentioned, the dosage of omega-3 may not have been enough to affect PA or relative weight. Instead of using standard doses, it is possible that the results would have been different if the dose was based on the body weight of each child. However, the dose was exactly the same as in the pilot study where an effect on PA was found. There is a need to further explore the interaction of PA and omega-3 fatty acids on different populations.

## Conclusion

Omega-3 supplementation was not found to affect PA or BMI SDS in healthy children. Children with lower initial levels of PA increased their PA more than children with higher initial levels. Thus, supplementation of omega-3 fatty acids could have the potential to increase PA in individuals with low levels of PA.

## Data Availability

The datasets generated during and/or analyzed during the current study are available from the corresponding author on reasonable request.

## References

[1] Cote AT, Harris KC, Panagiotopoulos C, Sandor GG, Devlin AM (2013). Childhood obesity and cardiovascular dysfunction. J Am Coll Cardiol..

[2] De Niet JE, Naiman DI (2011). Psychosocial aspects of childhood obesity. Minerva Pediatr..

[3] Danielsson P, Kowalski J, Ekblom O, Marcus C (2012). Response of severely obese children and adolescents to behavioral treatment. Arch Pediatr Adolesc Med..

[4] Hebestreit A (2014). Associations between energy intake, daily food intake and energy density of foods and BMI z-score in 2-9-year-old European children. Eur J Nutr..

[5] Pate RR (2013). Factors associated with development of excessive fatness in children and adolescents: a review of prospective studies. Obes Rev..

[6] Lavie CJ, De Schutter A, Milani RV (2015). Healthy obese versus unhealthy lean: the obesity paradox. Nat Rev Endocrinol..

[7] Ekelund U (2015). Physical activity and all-cause mortality across levels of overall and abdominal adiposity in European men and women: the European Prospective Investigation into Cancer and Nutrition Study (EPIC). Am J Clin Nutr..

[8] Harris KC, Kuramoto LK, Schulzer M, Retallack JE (2009). Effect of school-based physical activity interventions on body mass index in children: a meta-analysis. CMAJ..

[9] Calder PC (2015). Functional Roles of Fatty Acids and Their Effects on Human Health. JPEN J Parenter Enteral Nutr..

[10] Buckley JD, Howe PR (2010). Long-chain omega-3 polyunsaturated fatty acids may be beneficial for reducing obesity-a review. Nutrients..

[11] Pacifico L, Giansanti S, Gallozzi A, Chiesa C (2014). Long chain omega-3 polyunsaturated fatty acids in pediatric metabolic syndrome. Mini Rev Med Chem..

[12] Jeromson S, Gallagher IJ, Galloway SD, Hamilton DL (2015). Omega-3 Fatty Acids and Skeletal Muscle Health. Mar Drugs..

[13] Barber MD, Ross JA, Voss AC, Tisdale MJ, Fearon KC (1999). The effect of an oral nutritional supplement enriched with fish oil on weight-loss in patients with pancreatic cancer. Br J Cancer..

[14] Moses AW, Slater C, Preston T, Barber MD, Fearon KC (2004). Reduced total energy expenditure and physical activity in cachectic patients with pancreatic cancer can be modulated by an energy and protein dense oral supplement enriched with n-3 fatty acids. Br J Cancer..

[15] Karlberg J, Luo ZC, Albertsson-Wikland K (2001). Body mass index reference values (mean and SD) for Swedish children. Acta Paediatr..

[16] Cole TJ, Bellizzi MC, Flegal KM, Dietz WH (2000). Establishing a standard definition for child overweight and obesity worldwide: international survey. BMJ..

[17] Ekstedt M, Nyberg G, Ingre M, Ekblom O, Marcus C (2013). Sleep, physical activity and BMI in six to ten-year-old children measured by accelerometry: a cross-sectional study. Int J Behav Nutr Phys Act..

[18] Nyberg G, Nordenfelt A, Ekelund U, Marcus C (2009). Physical activity patterns measured by accelerometry in 6- to 10-yr-old children. Med Sci Sports Exerc..

[19] Pagels P (2014). A repeated measurement study investigating the impact of school outdoor environment upon physical activity across ages and seasons in Swedish second, fifth and eighth graders. BMC Public Health..

[20] Barnett AG (2005). Regression to the mean: what it is and how to deal with it. Int J Epidemiol..

[21] Bender N (2014). Fish or n3-PUFA intake and body composition: a systematic review and meta-analysis. Obes Rev..

[22] Buckley JD, Howe PR (2009). Anti-obesity effects of long-chain omega-3 polyunsaturated fatty acids. Obes Rev..

[23] Burrows T, Collins CE, Garg ML (2011). Omega-3 index, obesity and insulin resistance in children. Int J Pediatr Obes..

[24] Garemo M, Lenner RA, Strandvik B (2007). Swedish pre-school children eat too much junk food and sucrose. Acta Paediatr..

[25] Egert S, Stehle P (2011). Impact of n-3 fatty acids on endothelial function: results from human interventions studies. Curr Opin Clin Nutr Metab Care..

[26] Juarez-Lopez C, Klunder-Klunder M, Madrigal-Azcarate A, Flores-Huerta S (2013). Omega-3 polyunsaturated fatty acids reduce insulin resistance and triglycerides in obese children and adolescents. Pediatr Diabetes..

[27] Richardson AJ, Burton JR, Sewell RP, Spreckelsen TF, Montgomery P (2012). Docosahexaenoic acid for reading, cognition and behavior in children aged 7-9 years: a randomized, controlled trial (the DOLAB Study). Plos One..

[28] 2012 N. Nordic Nutrition Recommendations 2012. Nordic council of ministers 2013.

[29] Enghardt Barbieri, H., Pearson, M. & Becker, W. Riksmaten – barn. Livsmedels- och näringsintag bland barn i Sverige. National Food Agency, Sweden. *Uppsala* (2003).

[30] Lopez-Alarcon M, Martinez-Coronado A, Velarde-Castro O, Rendon-Macias E, Fernandez J (2011). Supplementation of n3 long-chain polyunsaturated fatty acid synergistically decreases insulin resistance with weight loss of obese prepubertal and pubertal children. Arch Med Res..

[31] de Ferranti SD (2014). Using high-dose omega-3 fatty acid supplements to lower triglyceride levels in 10- to 19-year-olds. Clin Pediatr (Phila)..

[32] Nobili V (2011). Docosahexaenoic acid supplementation decreases liver fat content in children with non-alcoholic fatty liver disease: double-blind randomised controlled clinical trial. Arch Dis Child..

[33] Christian, L. M. *et al*. Body weight affects ω-3 polyunsaturated fatty acid (PUFA) accumulation in youth following supplementation in post-hoc analyses of a randomized controlled trial. *PLoS One*. **5** (2017). 10.1371/journal.pone.0173087 PMC5381773 28379964

[34] Munro IA, Garg ML (2013). Prior supplementation with long chain omega-3 polyunsaturated fatty acids promotes weight loss in obese adults: a double-blinded randomised controlled trial. Food Funct..

